# Re-Evaluation of a Hyperendemic Focus of Metastrongyloid Lungworm Infections in Gastropod Intermediate Hosts in Southern Germany

**DOI:** 10.3390/pathogens14080800

**Published:** 2025-08-09

**Authors:** Alena Dusch, Lisa Segeritz, Judith Schmiedel, Anja Taubert, Carlos Hermosilla

**Affiliations:** 1 Institute of Parasitology, Biomedical Research Center Seltersberg (BFS), Justus Liebig University Giessen, 35392 Giessen, Germanycarlos.r.hermosilla@vetmed.uni-giessen.de (C.H.); 2 Institute of Medical Microbiology, Biomedical Research Center Seltersberg (BFS), Justus Liebig University Giessen, 35392 Giessen, Germany

**Keywords:** gastropod-borne disease, *Angiostrongylus vasorum*, lungworm, morphology, PCR, Germany

## Abstract

The metastrongyloid nematodes *Angiostrongylus vasorum*, *Aelurostrongylus abstrusus*, and *Crenosoma vulpis* can cause severe cardiopulmonary and respiratory symptoms in domestic dogs and cats and free-ranging canids and felids (e.g., foxes, wolves, wild cats, lynxes). Recent data on the prevalence of *A. vasorum* infections in dogs and foxes and on the prevalence of *Ae. abstrusus* and *Troglostrongylus brevior* infections in free-ranging lynxes and wild cats revealed several endemic and hyperendemic foci in Germany. Nonetheless, long-term investigations on the prevalence of metastrongyloid larvae infecting gastropod intermediate hosts are still scarce for Germany. To fill this gap, we conducted an epidemiological survey on native slugs and snails in a selected meadow close to Obrigheim, previously identified as a hyperendemic focus for canine angiostrongylosis. To re-evaluate this location as a ‘hotspot’ of canine angiostrongylosis, terrestrial slugs and snails (*n* = 533) were collected in all seasons, artificially digested, and microscopically and molecularly analyzed for the presence of metastrongyloid lungworm larvae. Here, the prevalence ranged greatly between seasons. In summer, 27.46% (59/215) of gastropods were infected with metastrongyloid larvae. In fall, the prevalence dropped to 10.00% (16/160) and lowest infection rates were observed in both winter (5.65%) and spring (1.47%). In total, *A. vasorum* was detected in 12.01% (64/533), *Crenosoma* sp. in 0.94% (5/533), and *Ae. abstrusus* in 0.38% (2/533) of gastropod samples. Even though total *A. vasorum* infection levels were revealed to be considerably lower than in the prior study, this epidemiological survey in principle reconfirms Obrigheim as a stable hyperendemic focus and thereby as a location with high metastrongyloid infection risk for domestic dogs, cats, and wildlife throughout the year. These results call for continuous epidemiological studies on gastropod populations to better understand metastrongyloid lungworm spread and infection dynamics over the years.

## 1. Introduction

Germany hosts multiple metastrongyloid lungworm species that infect different wildlife and domestic animals. Their life cycle is heteroxenous, with gastropods (i.e., snails and slugs) acting as obligatory intermediate hosts. The first-stage larvae (L1) are shed by the definitive host via its feces and are then taken up by the gastropod. In gastropods, the larvae develop to the third stage (L3). These enter the definitive host either by direct consumption of the gastropods or by accidental oral uptake after gastropod larval shedding [1,2]. As such, metastrongyloid lungworm infections are considered gastropod-borne diseases.

*Angiostrongylus vasorum*, also known as the French heartworm, is a parasite that lives in the right heart and the pulmonary arteries. It is considered the most pathogenic lungworm species in dogs. Clinical signs range from mild respiratory symptoms like coughing to severe respiratory distress, neurological disorder, hemorrhaging, and in some cases, even death [3,4,5]. This parasite does not only infect domestic dogs, but also foxes (*Vulpes vulpes*) [6,7], wolves (*Canis lupus*) [8,9], golden jackals (*Canis aureus*) [10,11], raccoon dogs (*Nyctereutes procyonoides*) [12], badgers (*Meles meles*) [13], and red pandas (*Ailurus fulgens*) [14]. Due to the rising urbanization of wild-living animals, especially synanthropic foxes, the life cycle easily shifts to domestic animals, such as dogs [7]. *A. vasorum* infections are occurring globally, being characterized by a patchy distribution of hyper- and hypoendemic foci in close proximity [3,15]. Recently, a ‘hotspot’ area was identified in 2018 in the city of Obrigheim within the Federal State of Baden-Wuerttemberg in Southern Germany reaching a prevalence of 62.96% in terrestrial gastropods [15].

*Crenosoma vulpis* is a metastrongyloid lungworm that infects both wildlife (e.g., foxes and wolves) and domestic canids. Adult parasites live in the bronchioles, bronchi, and trachea and lead to respiratory signs, such as acute coughing, dyspnea, bronchitis, and chronic cough [9,16,17,18,19]. Another important representative of this genus is *Crenosoma striatum*. Adult *C. striatum* nematodes are located in bronchi and pulmonary tissue of European hedgehogs (*Erinaceus europaeus*) [20]. European hedgehogs are omnivorous and consume terrestrial slugs/semi-slugs/snails on a regular basis, rendering them highly susceptible to this lungworm infection, which leads to respiratory disease, pneumonia, and even death in heavily *C. striatum*-infected animals [21,22].

*Aelurostrongylus abstrusus* is a cat lungworm that is distributed throughout Europe [15,23,24]. Similar to domestic cats, wild felid species, such as the Eurasian lynx (*Lynx lynx*) and the wild cat (*Felis silvestris*), are susceptible to infection. Thus, feline aelurostrongylosis may spread to domestic cats and vice versa. Symptoms include respiratory distress, coughing, sneezing, and respiratory discharge [25,26].

*Troglostrongylus brevior* is another cat lungworm present in Germany. It is less often described, even though its relevance seems to be increasing. Feline troglostrongylosis causes mild to severe respiratory disease, which may lead to death in young cats [27,28]. This parasite was recently found in German Eurasian lynxes [29,30] and wildcats [31], suggesting the chance of lungworm transmission between free-living and domestics cats.

Metastrongyloid lungworm infections are spreading worldwide, expanding from regions in which they were formerly endemic [7]. Prevalence studies on lungworm infections in both free-living and domestic definitive hosts were performed in Germany, Denmark, the United Kingdom, France, and Italy, among others [7,8,18,32]. These studies mostly confirmed a classical patchy distribution of lungworm infections in hyperendemic foci, so-called hotspots [3,15]. Recent data suggest that lungworms, especially *A. vasorum*, seem to spread from ‘hotspots’ into new areas where no lungworm infections were previously recorded [7,33,34]. This dynamic spread calls for further studies on both well-known hyperendemic foci and potential new ‘hotspots’ as recently performed for Colombia in South America [35,36,37].

Metastrongyloid lungworm infections represent under-recognized diseases in both researchers and practicing veterinarians despite their worldwide prevalence [7,38]. While there are numerous studies on prevalences and clinical manifestations of affected definitive hosts in Europe, studies on the prevalence and development of lungworm larvae in gastropod intermediate hosts are highly neglected.

Aim of the current study was to reconfirm an *A. vasorum* hyperendemic focus in Obrigheim, and to identify environmental and biological factors associated with this particular region. Special attention was paid to potential seasonal changes in prevalence of metastrongyloid infections in gastropod intermediate hosts. Furthermore, we intended to inform local veterinarians and dog owners on transmission patterns of canine angiostrongylosis in Obrigheim.

## 2. Materials and Methods

### 2.1. Sample Collection

The sampling area of Obrigheim (Baden-Wuerttemberg, Germany) was chosen according to previous published data of lungworm infections in both definitive [39] and intermediate hosts [15]. Thus, a special meadow, frequented by dog walkers and located close to town, was studied again due to its ideal conditions for intermediate host habitat and potential infection area of domestic as well as wild definitive hosts.

Gastropod collection was performed once in all four seasons (spring, summer, autumn, and winter). Please refer to Supplementary Figure S1 for details about sampling conditions. A total number of 533 gastropods (i.e., snails, slugs) including 11 different gastropod species were collected on the hotspot meadow in 2022 and 2023. Representative images of some identified species are shown in Supplementary Figure S2. Since gastropod activity spikes in the early morning hours, the sampling was performed around sunrise. Depending on the weather conditions, gastropod collection required between 1 and 4 h.

Under current German national law, no permission is needed for gastropod collection. Additionally, in April of 2023, water samples (1 L) were collected, since the meadow was flooded due to heavy rain.

### 2.2. Gastropod Procession

To achieve cryo-euthanasia, gastropods were frozen at −20 °C and thereafter preserved at 4 °C until further procession. Each gastropod was weighed and morphologically identified before being submitted to artificial digestion according to Lange et al. [40]. Thereafter, the remnants were sieved, and all 533 samples were individually analyzed by light microscopy (Olympus BH-2).

### 2.3. Microscopical Analysis

**Figure 1:**
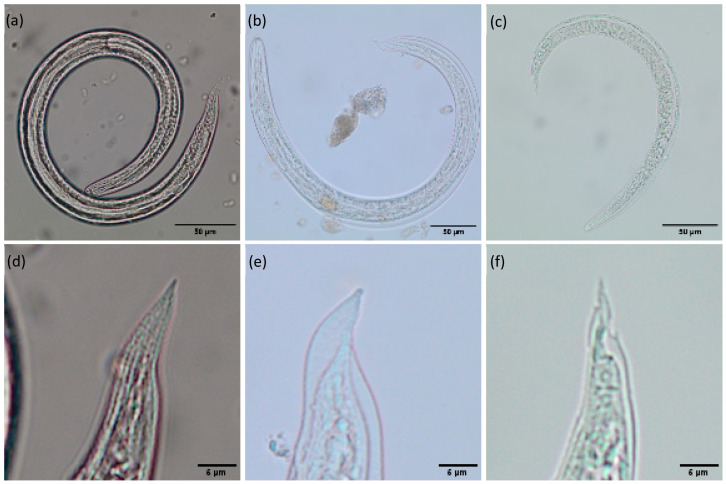
Morphology of metastrongyloid lungworm larvae. (**a**) *Crenosoma* sp. L3; (**b**) *Angiostrongylus vasorum* L3; (**c**) *Angiostrongylus vasorum* L1; (**d**) *Crenosoma* sp. L3 tail; (**e**) *Angiostrongylus vasorum* L3 tail; (**f**) *Angiostrongylus vasorum* L1 tail.

Metastrongyloid lungworm larvae were identified according to their morphometric characteristics, such as larval length and width, esophagus length and type (non-rhabditiform, one third to one half of larval length), as well as tail morphology [41]. By this way, lungworm larvae species can be differentiated from each other as well as from possibly present free-living larvae or gastropod parasitic nematodes [40]. Please refer to Figure 1.

### 2.4. DNA Extraction from Larvae

DNA extraction was performed on larvae collected from 10 gastropods containing high larval burdens in order to assess the applicability of molecular techniques in species identification. Following microscopic analysis, all the larvae found in one individual snail or slug were pooled, thus creating 10 samples for further processing. The DNA of the pooled larvae was then isolated using two different extraction methods.

The first method used was the DNeasy Blood & Tissue Kit (Qiagen) and was carried out according to the manufacturer’s instructions.

The second method involved using the EMAG (bioMérieux) automatic nucleic acid extraction system. A customized protocol for extracting DNA from tissue was employed. In brief, the larvae were homogenized by bead beating and transferred to a new vessel containing a 1:1 dilution of lysis buffer containing guanidine thiocyanate. After a short centrifugation step, the supernatant was loaded into the EMAG. After extraction, the DNA was eluted in Aqua dest.

### 2.5. Molecular Identification of Lungworm Species

PCR was performed on the 10 highly positive samples using the nematode forward primer NC1 5′-ACGTCTGGTTCAGGGTTGTT-3′ and the reverse primers NC2 5′-TTAGTTTCTTTTCCTCCGCT-3′ and MetR 5′-CCGCTAAATGATATGCTTA-3′ [15,42,43]. The expected amplicon size was approximately 300 base pairs, targeting the ribosomal RNA (rRNA). Subsequently, 10 samples were sent to LGC Genomics, Berlin, Germany for sequencing. Thereafter, the samples were compared with deposited gene samples in GenBank via BLAST (https://blast.ncbi.nlm.nih.gov/Blast.cgi, accessed on 10 July 2025)

### 2.6. Information for Veterinary Practices and Pet Owners

In order to spread awareness among pet owners walking their dogs in or near the hyperendemic focus, an information sign was mounted. The sign contained general information on lungworms, ways of infection, and possible symptoms. A website was created that could simply be accessed by scanning a QR code with a mobile device. The website included a questionnaire for dog owners posing the following questions: age, sex, and breed of the dog, potential stays abroad, anthelmintic status and regimen, diagnoses of lungworms, and related symptoms.

## 3. Results

In all four seasons, gastropods proved positive for metastrongyloid lungworm larvae with highly variable season-dependent prevalence. A total of 78/533 (14.63%) gastropods were infected. *A. vasorum* was detected in 12.01% (64/533), *Crenosoma* sp. in 0.94% (5/533), and *Ae. abstrusus* in 0.38% (2/533) of gastropod samples. In summer, 27.46% (59/215) of gastropods were infected with metastrongyloid larvae. In fall, the prevalence dropped to 10.00% (16/160) and lowest infection rates were observed in both winter (5.65%) and spring (1.47%). In total, *A. vasorum* was detected in 12.01% (64/533), *Crenosoma* sp. in 0.94% (5/533), and *Ae. abstrusus* in 0.38% (2/533) of gastropod samples.

PCR was performed on a total of 10 of the infected gastropods in order to check PCR functionality as well as confirm microscopy results. *Crenosoma* sp. was confirmed in four, *A. vasorum* in three, *Ae. abstrusus* in two, and *C. striatum* in a single sample.

**Figure 2:**
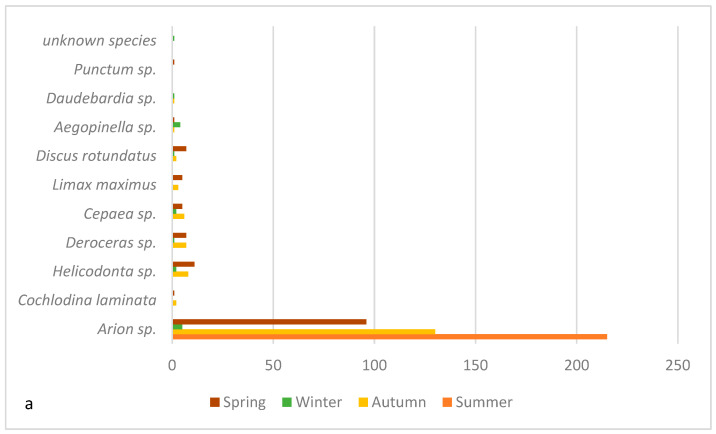

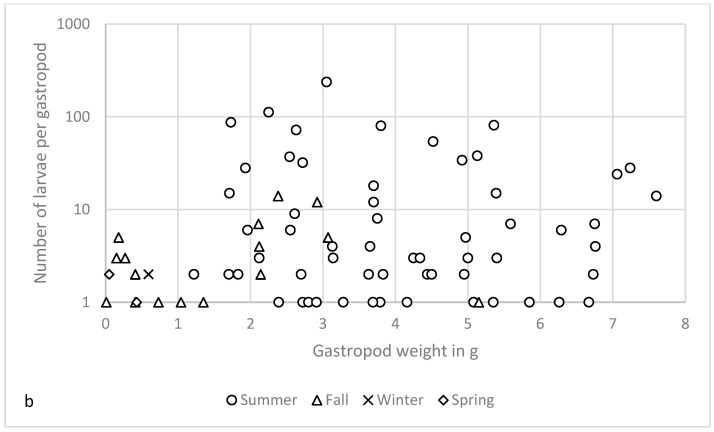
(**a**) Terrestrial gastropod species collected throughout the seasons; (**b**) correlation of gastropod weight and larval burden (log 10 scale).

Overall, mostly Spanish slugs (*Arion* sp.) were discovered in the meadow (Figure 2). The weight of the gastropods ranged from 0.01 g to 10.11 g and showed an average weight of 4.02 g. In principle, a high prevalence of *A. vasorum* infection was re-confirmed in gastropods.

In summer, a total of 27.46% (59/215) of gastropods were infected with metastrongyloid larvae; 22.79% (49/215) of slug samples proved positive for *A. vasorum* larvae (Table 1). Besides *A. vasorum*, *Crenosoma* sp. larvae were also detected (3/215) but at a much lower prevalence (1.40%). In addition, one slug was found positive for *Ae. abstrusus*. The larval burden (number of larvae observed in one individual gastropod) ranged from one to 241 larvae per slug (Figure 2).

In fall, metastrongyloid lungworm species prevalence was considerably lower (Table 1). In total, 10% (16/160) of gastropod samples were positive for metastrongyloid lungworm larvae. *A. vasorum* larvae were diagnosed in 14 slugs, while only 2 slugs were found positive for *Crenosoma* sp. larvae, thus reaching prevalences of 8.7% and 1.2%, respectively. Additionally, *Ae. abstrusus* was observed in a single slug (1/160, 0.6%). In this season, a greater variety of gastropod species was collected (please refer to Figure 2a). While majority of infected gastropods belonged to *Arion* sp., a single leopard slug (*Limax maximus*) was also found positive for metastrongyloid lungworm larvae. The larval burden ranged from 1 to 14 larvae per slug. In addition to the prevalence and the larval burden lower, the average snail weight proved lower when compared to summer with an average gastropod weight of 0.7 g. In this season, a double infection was observed. One *Arion* sp. was infected by both *A. vasorum* and *C. striatum.*

In winter, snow was covering the meadow and gastropods were collected by digging through the snow close to trees and a pile of wood. Given these challenging conditions, a considerably lower number of gastropods (*n* = 18) with a great species diversity were collected. Here, a metastrongyloid lungworm larva was detected only in a single *Deroceras* sp. slug, thus indicating a lungworm prevalence of 5.56%. Unfortunately, the species of this larva could not be identified microscopically. Thus, the larval burden was very low. The average weight of collected gastropods in winter was low as well with 0.6 g.

In spring, a total of 136 gastropods of nine different species were collected at the hotspot meadow. Two of these gastropods were found infected with metastrongyloid lungworm larvae (2/136, 1.48%). Thus, a single *Arion* sp. slug proved infected with metastrongyloid larvae that could not be identified. One *Helicodonta* sp. snail contained a single *A. vasorum* L3. Hence, the larval burden in spring ranged from one to three larvae per gastropod. The gastropods collected in spring were light, with an average weight of 0.45 g. Of note, one gastropod was co-infected with two lungworm species, i.e., *A. vasorum* and *C. striatum*. Triple infections, as well as co-infections with *Ae. abstrusus* and *C. vulpis* were not observed.

The larval burden was highest in summer, with one slug carrying a mean of 77.07 larvae per g bodyweight (BW). The lowest burden was observed in summer as well, with 0.15 larvae per g BW. There was no clear correlation between gastropod BW and larval burden (Figure 2). Most gastropods (56/77) harbored a relatively low larval burden with less than 10 larvae per individual. Very high numbers of larvae (more than 100) were discovered only in two gastropods.

Microscopic species determination in the 10 highly infected gastropods was confirmed by PCR and sequencing (Table 2) according to Segeritz et al. and Ash et al. [15,41]. No differences between the microscopic analysis and PCR was observed.

The analysis of the water collected in spring revealed the presence of a single metastrongyloid lungworm larva.

Three veterinary practices around the city of Obrigheim were called and informed about the hyperendemic area but showed little to no interest. The questionnaire linked to the sign mounted on the meadow was filled out by a total of nine pet owners, but none of them reported on a history of lungworm infections in their dogs.

## 4. Discussion

This study reconfirms Obrigheim as a hyperendemic focus for *A. vasorum* as well as other lungworm species infections in gastropod intermediate hosts as previously reported [15]. The reasons for this meadow being a ‘hotspot’ area may be influenced by various environmental/ecological factors, such as diverse flora (e.g., grassland, trees, fungi, and bushes) and fauna (i.e., mollusks, arthropods, amphibians, reptiles, birds, rodents, and beavers), a stream as water body, and ideal humid climatic conditions throughout the year. Even in very hot and dry summer conditions (i.e., in August 2022), we observed that the meadow still was lush and green. A small stream, called Heiligenbach, runs through the meadow and is surrounded by trees. The stream even has a dam constructed by native beavers. A larger forested area is also located not far from this meadow. All these geographical and ecological conditions also render this meadow ideal for wildlife. As mentioned before, wild canids and wild felids might play a role in the transmission of lungworms to domestic animals [13,31]. While wild cats and Eurasian lynxes are not known to reside nearby, foxes are prevalent all throughout Germany, including Obrigheim. Synanthropic foxes might also defecate on this meadow, thereby infecting gastropods, and the life cycle may spill over to domestic dogs. During sampling, many dog owners were observed walking their dogs on the small path nearby and in the meadow itself.

This study highlights the extreme fluctuating lungworm prevalence in gastropod intermediate hosts. The prevalence of lungworm larvae in gastropods not only varies drastically in different seasons but also fluctuates between the years. A total gastropod lungworm prevalence of 27.46% was observed in summer. This stands in contrast to the previous study by Segeritz et al. [15], which documented a total prevalence of 17.6% during the summer months. In 2018, the highest prevalence in gastropods was observed in fall with 75.93% [15], thereby contrasting current findings in the same season of 2022, where the total prevalence was only 10%. The higher prevalence in the summer of 2022 may be linked to a different collection time as well as gastropod species collected. Based on the life cycle of *Arion* sp., it is quite logical to find larger and heavier slugs in the summer months. In this season, these slug species tend to reach their maximal length and weight before mating, laying eggs and dying in the late summer months [44,45,46]. Even though it is possible for multiple slug generations to be formed within a year, a single-generation cycle is more common. Thus, the slugs collected in fall either represented a second and smaller adult generation or the newly hatched juvenile slugs. Of note, most gastropods collected in summer were large *Arion* sp. slugs. Their weight ranged from 0.01 g to 10.11 g with an average weight of 4.02 g. As suggested in previous studies [47,48], there may be a correlation between gastropod maturity and size and even burden of infection. While we were unable to confirm the correlation between gastropod weight and burden of infection, it is quite likely that older gastropods are more prone to being infected, since larger slugs feed on a larger feces volume than smaller slugs, and thus the likelihood of infection may increase. In fall, the gastropods collected and analyzed were generally smaller. The average weight of these gastropods was 0.7 g. The *Arion* sp. slugs collected in October most likely represented, due to their small size, the new generation that hatched later in the year. As such, they did not consume as much feces as their older and larger counterparts. In addition, higher species diversity was observed in fall. This should also be taken into account while comparing the data between the years 2018 and 2022. During winter, *Arion* sp. are known to survive through the juvenile life stages. Other gastropod species, such as the white-lipped snail *Cepaea hortensis*, survive even freezing temperatures by adjusting their metabolic rate [49]. As such, metastrongyloid lungworm larvae are also able to survive the winter inside gastropod intermediate hosts and thereby preserve hyperendemic focus until the gastropods become more active in spring again. As such, the persistence of the life cycle of lungworm infections is possible throughout the seasons and leads to a new cycle in the next year, as reported elsewhere [50,51].

Furthermore, temperature and rainfall play a pivotal role in both survival and multiplication of snails, semi-slugs, and slugs. The summer of 2018 in Germany was extremely hot and dry [52], which influenced both flora and fauna. In these hot and dry climatic conditions, snails and slugs are less active in both reproduction and mobility. These ecological conditions may explain the higher lungworm prevalence in summer of 2022. The lower prevalence in fall of 2022 may also be explained by the higher species diversity. Generally speaking, as mentioned before, *Arion* sp. slugs are described as highly suitable intermediate hosts for metastrongyloid lungworm species. The largest numbers of both collected and infected gastropods were indeed *Arion* sp. This might be linked to multiple factors, such as a potential higher prevalence of those slugs compared to other gastropod species in Obrigheim, or simply by their morphology, which facilitated their visual detection, due to their orange color and size. These invasive slugs are also reported as spreading worldwide. This slug species was reported to be transported passively, for example, in animal fur, clothing, and vehicles [45], and thus to transfer endogenous lungworms, acquiring new habitats. In general, *Arion* sp. were previously described as suitable obligate intermediate hosts for lungworm larvae [2,40]. During field gastropod collections, *Arion* sp. were frequently found feeding on dog feces thereby confirming coprophagic activities [53]. Other gastropods are less likely to feed on feces, since they prefer other feed, such as a variety of plants, fungi, and seedlings, as well as mycelium [54,55].

Furthermore, the invasive slug *Arion vulgaris* may facilitate parasite spillover from wildlife to domestic animals by increasing environmental contamination with infective larvae. A high abundance of *A*. *vulgaris* may also cause parasite transmission and facilitate spillback by increasing endemic lungworm infections re-entering wildlife populations. Alternatively, having a range of non-competent or less suitable gastropod hosts present, along with *A*. *vulgaris*, may have dilution effect to reduce transmission pressure.

In this study, we also found *L. maximus*, *Helicodonta* sp., and *Deroceras* sp. positive for lungworm larvae, even though at low numbers. The slug *L. maximus* is known for cannibalism [56], and the possibility should not be overlooked that this slug species might become infected with metastrongyloid lungworms by feeding on other infected snails or slugs. The number of infected slugs may even increase in this theory when multiple slugs feed on a single infected carcass.

Another important factor to consider in changing prevalences is the behavior of the definitive hosts and pet owners. During cold or rainy periods, they are less likely to be walked in the meadow. In summer months with warm and sunny conditions, pet owners are more likely to go outside for longer walks with their dogs on and/or around this area. Since the prevalence in summer was observed to be higher than in other seasons, the likelihood of both definitive and thus also intermediate host infection is increasing in summer. Not only is it possible for dogs to consume gastropods willingly, but they may also engorge smaller slugs and snails accidentally, for example, while eating grass or feeding on rotten fruits and vegetables. The smallest infected slug we found weighed only 0.01 g. As such, it seems very feasible that a dog or a cat might ingest such tiny gastropods purely by accident. The hunting and consuming of paratenic hosts, such as rodents, reptiles, birds, and frogs, is also an important way of infection, particularly for feline aelurostrongylosis [57,58]. Multiple frogs were observed during the summer sample collection. In addition, while sampling at the meadow, we found dog feces in multiple locations in all seasons. This lack of hygiene by dog owners might additionally contribute to the persistence of this hyperendemic focus in Obrigheim. Since German dog owners travel frequently with their pets, patent lungworm infections may be spilled-over by infected animals into new areas where lungworm infections were not yet prevalent.

Even though we retrieved only a few responses to the questionnaire, it is interesting to notice that none of the pet owners reported on lungworm infections in their dogs. This might be due to multiple reasons. Lungworm infections are not a primary focus in veterinary practices and clinics. Additionally, in order to detect lungworm larvae, a Baermann–Wetzel apparatus needs to be used, since they cannot be detected via flotation in most cases [59]. Furthermore, many lungworm infections are subclinical and with intermittent larval excretion. Moreover, in case of clinical signs like apathy, lethargy, coughing, or gastrointestinal symptoms, these may be unspecific and eventually not be linked to lungworm infections [3,4]. In addition, it is still common practice in Germany to treat dogs with anthelmintics on a regular basis without verifying a potential parasitic infection, for example, once every three months. Commonly used anthelmintic drugs, such as fenbendazole and moxidectin, are effective against multiple species of endoparasites, lungworms included [60,61]. As a result of periodical treatments with endoparasiticides, lungworm infections may be reduced. Nevertheless, it is of utmost importance to raise awareness to potential lungworm infections in both veterinary practitioners and pet owners.

The finding of a single metastrongyloid lungworm larva in the water collected at the meadow indicated that larval survival in water seems in principle possible. It has been described for the zoonotic *Angiostrongylus cantonensis* to actively leave deceased gastropod intermediate hosts and to survive exogenously as L3 for some weeks [15,39,62]. This L3 survival capacity reveals most likely novel transmission routes for definitive hosts by drinking water from a puddle with a dead but metastrongyloid-infected gastropod.

In this study, PCR was used as an alternative method for metastrongyloid lungworm detection; however, its limitations need to be considered. PCR from digested snail tissue is generally challenging due to various inhibitors, such as mucine and digestive enzymes (endonucleases, proteases) as well as the digestive solution itself [40,63]. Bacteria present in the snail’s mucus can also inhibit PCR due to cell wall fragments and nucleases [64]. In addition, low parasite burdens may further complicate the procedure, since the sample can be easily lost during DNA extraction. As such, the PCR technique may not be sensitive enough to detect infections if the parasite burden is too low. Furthermore, no conclusions can be drawn on the parasitic burden, the vitality of the larvae, or the successful development into L3 larval stages.

## 5. Conclusions

This study reconfirms Obrigheim as a stable hyperendemic focus of Southern Germany for metastrongyloid lungworm infections in gastropods. These epidemiological findings suggest that environmental factors may play a crucial role in the varying prevalence throughout the year. Further research is required to analyze the influences of climatic change and other conditions on the prevalence of lungworm larvae in gastropods and thus the infection risk for definitive hosts.

## Figures and Tables

**Table 1:** Metastrongyloid lungworm species prevalences in terrestrial gastropods.

	Spring	Summer	Fall	Winter
*A. vasorum*	0.74%	22.79%	8.7%	0%
*Crenosoma* sp.	0%	1.40%	1.2%	0%
*Ae. abstrusus*	0%	0.47%	0.6%	0%
Metastrongyloidea	0.74%	2.80%	0%	5.56%
Total	1.48%	27.46%	10%	5.56%

**Table 2:** Molecular identification of lungworm larvae species from Obrigheim.

Species	Accession number	Season
*A. vasorum*	PV917150	Summer
*Crenosoma* sp.	PV917152	Summer
*A. vasorum*	PV917158	Summer
*Ae. abstrusus*	PV917161	Summer
*A. vasorum*	PV917159	Summer
*C. striatum*	PV917154	Summer
*Crenosoma* sp.	PV917156	Summer
*Ae. abstrusus*	PV917171	Fall
*Crenosoma* sp.	PV917167	Fall
*Crenosoma* sp.	PV917170	Fall

## Data Availability

Data are contained in this article.

## Supplementary Materials

The following supporting information can be downloaded at: https://www.mdpi.com/article/10.3390/pathogens14080800/s1, Figure S1: Sampling conditions throughout the year (a) Summer; (b) Fall; (c) Winter; (d) Spring, flooded meadow. Figure S2: Gastropod species observed in Obrigheim (a) *Limax maximus*; (b) *Helicodonta obvoluta*; (c) *Cepaea* sp.; (d) *Arion* sp. (juvenile).
