# Supplementary material for: Re-Evaluation of a Hyperendemic Focus of Metastrongyloid Lungworm Infections in Gastropod Intermediate Hosts in Southern Germany
Source: Pathogens. 2025 Aug 9;14(8):800. doi: 10.3390/pathogens14080800 (PMC12389336; doi:10.3390/pathogens14080800)
Supplement: Supplementary file 1 [file pathogens-14-00800-s001.zip › pathogens-3766910-supplementary.pptx]

## Slide 1
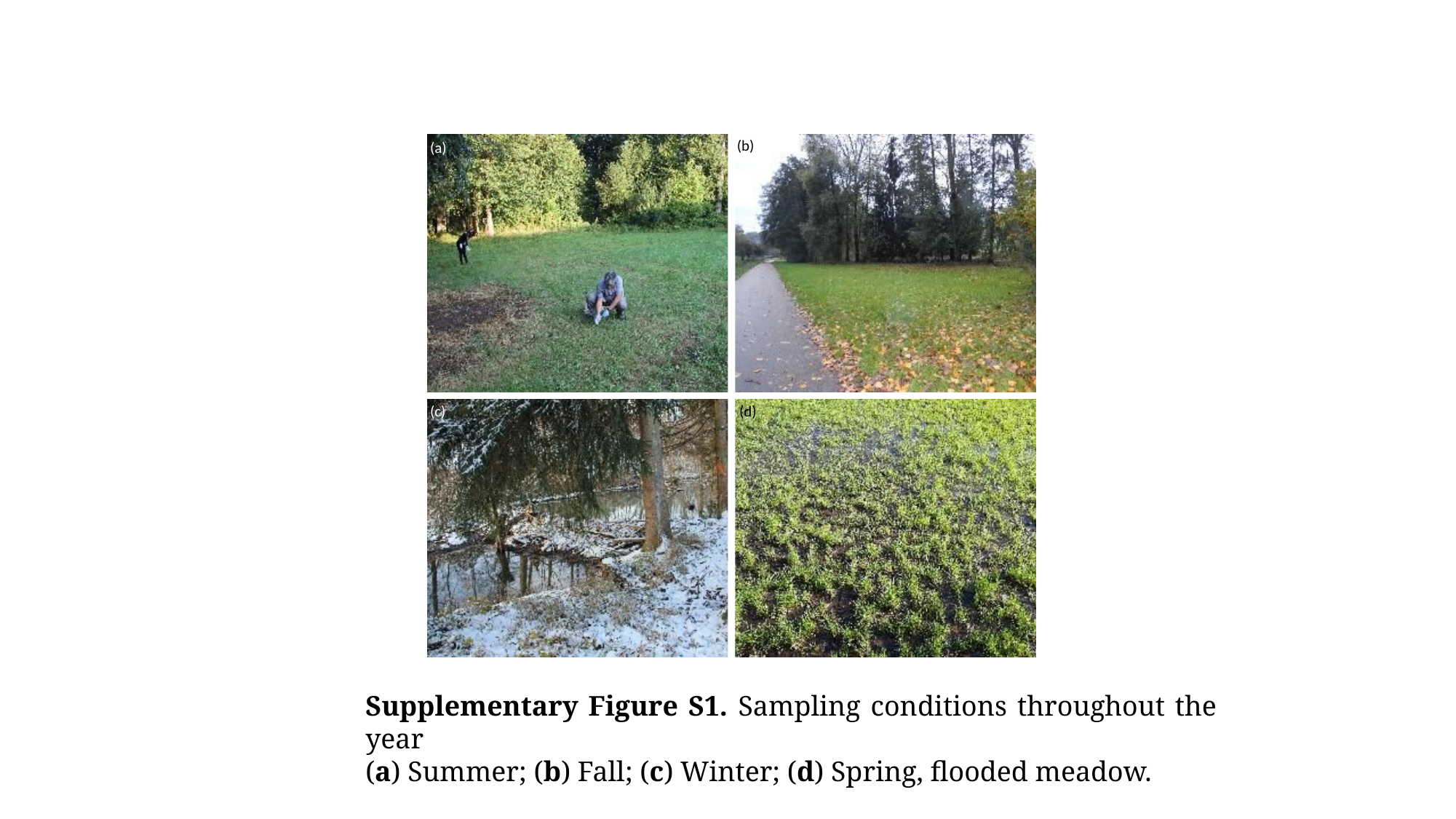

(b)
(a)
(c)
(d)
Supplementary Figure S1. Sampling conditions throughout the year
(a) Summer; (b) Fall; (c) Winter; (d) Spring, flooded meadow.

## Slide 2
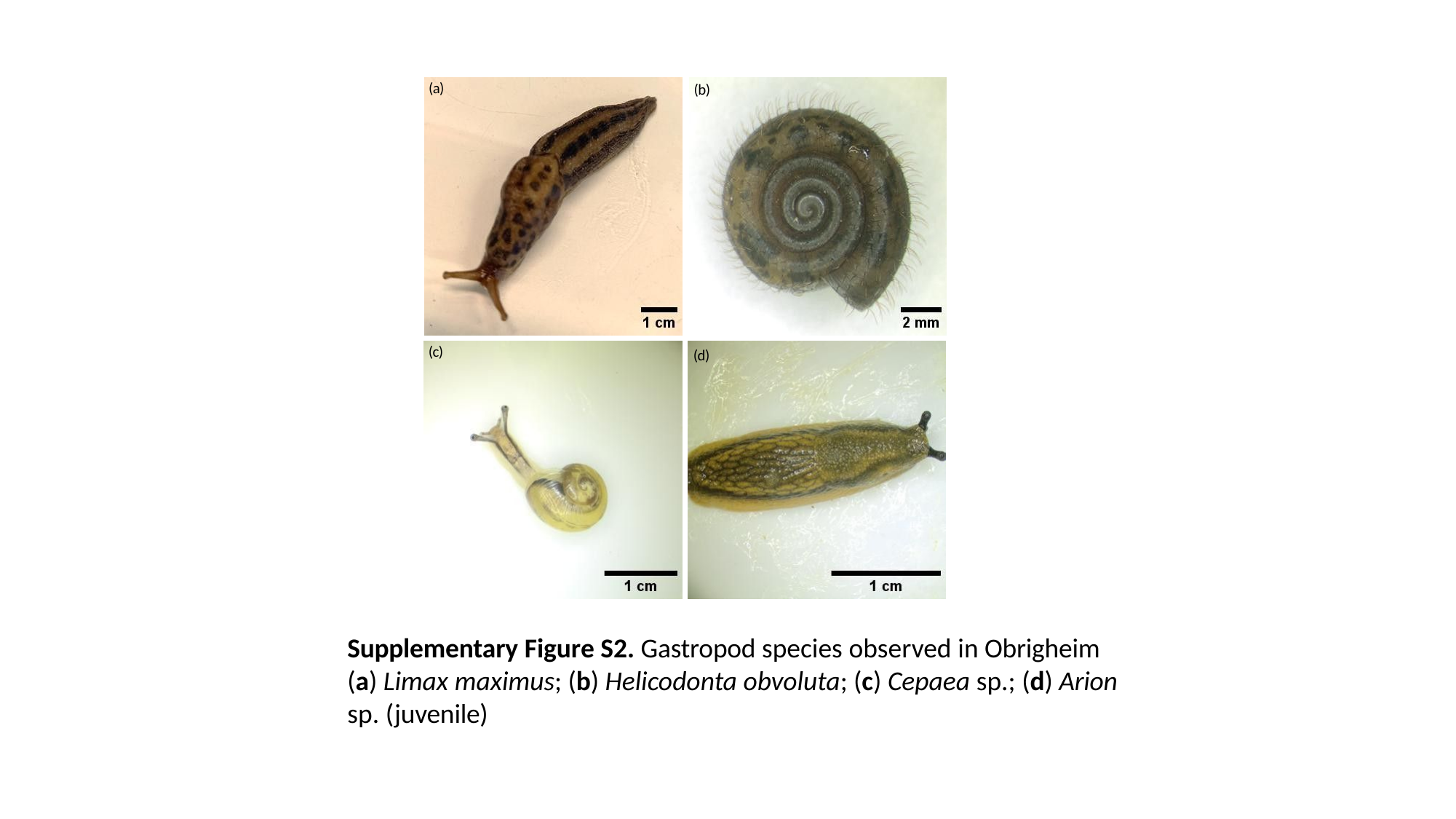

(a)
(b)
(c)
(d)
Supplementary Figure S2. Gastropod species observed in Obrigheim
(a) Limax maximus; (b) Helicodonta obvoluta; (c) Cepaea sp.; (d) Arion
sp. (juvenile)
